# Measuring Daily Activity Rhythms in Young Adults at Risk of Affective Instability Using Passively Collected Smartphone Data: Observational Study

**DOI:** 10.2196/33890

**Published:** 2022-09-14

**Authors:** Benny Ren, Cedric Huchuan Xia, Philip Gehrman, Ian Barnett, Theodore Satterthwaite

**Affiliations:** 1 Department of Biostatistics, Epidemiology, and Informatics University of Pennsylvania Perelman School of Medicine Philadelphia, PA United States; 2 Penn Lifespan Informatics and Neuroimaging Center University of Pennsylvania Perelman School of Medicine Philadelphia, PA United States; 3 Department of Psychiatry University of Pennsylvania Perelman School of Medicine Philadelphia, PA United States; 4 Michael J Crescenz VA Medical Center Philadelphia, PA United States

**Keywords:** mobile health, mHealth, hidden Markov model, mental health, circadian rhythm, mobile phone

## Abstract

**Background:**

Irregularities in circadian rhythms have been associated with adverse health outcomes. The regularity of rhythms can be quantified using passively collected smartphone data to provide clinically relevant biomarkers of routine.

**Objective:**

This study aims to develop a metric to quantify the regularity of activity rhythms and explore the relationship between routine and mood, as well as demographic covariates, in an outpatient psychiatric cohort.

**Methods:**

Passively sensed smartphone data from a cohort of 38 young adults from the Penn or Children’s Hospital of Philadelphia Lifespan Brain Institute and Outpatient Psychiatry Clinic at the University of Pennsylvania were fitted with 2-state continuous-time hidden Markov models representing active and resting states. The regularity of routine was modeled as the hour-of-the-day random effects on the probability of state transition (ie, the association between the hour-of-the-day and state membership). A regularity score, Activity Rhythm Metric, was calculated from the continuous-time hidden Markov models and regressed on clinical and demographic covariates.

**Results:**

Regular activity rhythms were associated with longer sleep durations (*P*=.009), older age (*P*=.001), and mood (*P*=.049).

**Conclusions:**

Passively sensed Activity Rhythm Metrics are an alternative to existing metrics but do not require burdensome survey-based assessments. Low-burden, passively sensed metrics based on smartphone data are promising and scalable alternatives to traditional measurements.

## Introduction

### Background

The proliferation of smartphone use in mobile health (mHealth) research has resulted in a wealth of longitudinal data capable of quantifying human behaviors pertinent to the study of mental health [1-3]. Smartphones collect a wide variety of sensor data, ranging from accelerometer and geolocation (ie, GPS) data to screen time and social interactions, which are increasingly being used as digital biomarkers of behavior in a variety of contexts [4-7]. The continuous collection of this wealth of data enables us to study an individual’s pattern of behavior across the course of each day. Many behaviors show a diurnal rhythm, an observed 24-hour periodic pattern, some of which are measurable through digital biomarker data [6,8]. These rhythms reflect endogenous physiological circadian processes related to many clinically relevant outcomes [9]. A wide range of physiological processes follows a circadian rhythm [10-12], such as cardiometabolic function and gene expression [13-15]. Certain immunological processes and drug efficacy are sensitive to specific points in the circadian cycle, highlighting the need to understand the role of circadian rhythms from a pharmacodynamics perspective [16,17]. In addition, disruptions in rest-activity cycles have been associated with adverse outcomes in posttraumatic stress and affective disorder studies [18,19]. This underscores the need to meaningfully quantify circadian rhythms in ecological contexts, such as the assessment of diurnal rhythms, as reflected in smartphone use data.

Before mobile devices were used to gather high-frequency ecological momentary assessment (EMA) and continuous streams of sensor data collection, information obtained from diaries and surveys was used for scoring the regularity of diurnal activities [20,21]. For example, Social Rhythm Metric (SRM) uses daily administered diaries to record the timing of routine activities (eg, getting out of bed, eating lunch, and starting work). To score highly on the SRM, one must consistently perform these activities close to the same time of day for most records, such as consistently waking up at the same time every day. Subsequently, high scores can be interpreted as greater regularity in routine or rhythm and provide a useful quantification of the regularity of rhythms, which can be used to study clinical outcomes. Disruptions to the regularity of rhythms have been associated with psychiatric disorders (eg, bipolar disorders [22-24], anxiety disorders [25], depression [12,26], mood or affective disorders [27,28], posttraumatic stress disorder [27,29], and substance-related disorders [30,31]). Measures of regularity are often markers of treatment efficacy in social rhythm therapies aimed at improving mental health [27]. Despite the utility of diary-based scores in the studies of psychopathology, manual data collection could be burdensome, susceptible to self-report bias, recall bias, nonresponse bias, and experience degradation over time because of the loss of engagement with the participant [1,32-34].

The potential use of smartphones for long-term data collection is made possible by ensuring a low burden on the participant, particularly through sensor data that can be collected passively (ie, without requiring active participation or input from the user) [35,36]. As irregular circadian rhythms are associated with a range of psychiatric disorders, particularly affective disorders [28,37,38], the logical next step is to use smartphone sensor data to quantify regularity in diurnal rhythms and then identify correlated clinically relevant outcomes [4]. If passively collected smartphone sensor data can be used to inform clinically relevant behaviors and symptoms, this would provide an effective and low-burden approach to mental health assessment and treatment monitoring [39].

### Objectives

Many models have been proposed to study circadian or diurnal rhythms based on passively collected sensor data, ranging from simple rule-based analyses to deep learning models [18,40-42]. Longitudinal data collected in mHealth studies can be viewed as a multivariate time series and subsequently often draw upon a variety of longitudinal and stochastic models [43-46]. A focal point of mHealth studies is to model rest-activity cycles or other categorical outcomes [47-50]. Modeling the dichotomy of rest-active states often simplifies clustering [8,51,52] or classification [5,52,53] problems where rhythmic effects can be modeled with harmonics [45,54-56]. For our purposes, to model transitions between different circadian biological states over the course of the day, we modeled these data using hidden Markov models (HMMs). In this framework, we captured the circadian nature of behaviors, as measured using smartphone sensor data, through the use of hour-of-the-day random effects [57-59].

Despite the potential of mHealth data, statistical models that translate these data into interpretable measures of diurnal rhythms and markers to manage mental health are an active area of research [27,39]. In the time series literature, random effects have been used to model seasonality or other periodic effects [59,60]. Our continuous-time HMM (CT-HMM) transitions between rest-activity cycles use individual-specific random intercepts for hours of the day (eg, 12 AM to 1 AM and 1 AM to 2 AM) to allow for personalized patterns of diurnal activity [61]. By fitting this model to each individual separately, we were able to quantify the regularity of activity rhythms or routines and determine how this strength of routine is correlated with a variety of demographic and clinical outcomes. Finally, we developed a novel score to gauge the regularity of activity rhythms and determine how this score correlates with self-reported sleep-related outcomes and other characteristics in a sample of adolescents with or at risk of affective instability.

## Methods

### Participants

A sample of 41 adolescents and young adults (28/41, 68% female participants) aged 17 to 30 (mean 23.4, SD 3.5) years were enrolled as part of a study on affective instability in youth. Participants were recruited via the Penn or Children’s Hospital of Philadelphia Lifespan Brain Institute or through the Outpatient Psychiatry Clinic at the University of Pennsylvania [62]. Of these 41 participants, 38 (92%) met the criteria for an Axis I psychiatric diagnosis based on a semistructured clinical interview, and 33 (80%) met the criteria for >1 disorder [63]. In addition, 39% (16/41) of participants met the criteria for a personality disorder based on an assessment with the Structured Clinical Interview for the Diagnostic and Statistical Manual of Mental Disorders-4 Axis II Personality Disorders (Tables 1 and 2) [63]. As a secondary analysis, we used data collected from a prior study. Subsequently, the availability of new clinical data was a limitation of our retrospective study design. Although we had digital EMA and passive sensor data for all participants, baseline measurements such as the Pittsburgh Sleep Quality Index (PSQI) and psychiatric diagnoses were not available for all participants.

**Table 1:** Psychiatric diagnoses of participants (N=41).

Diagnosis			Participants, n (%)
**Axis I diagnosis**			
	No diagnosis		3 (7)
	**Diagnosis**		38 (93)
		Major depressive disorder	24 (59)
		Bipolar disorder	4 (10)
		Depressive disorder NOS^a^	1 (2)
		Mood disorder NOS	1 (2)
		Generalized anxiety disorder	14 (34)
		Posttraumatic stress disorder	14 (34)
		Social phobia	12 (29)
		Obsessive-compulsive disorder	11 (27)
		Panic disorder	5 (12)
		Anxiety disorder NOS	2 (5)
		Attention-deficit or hyperactivity disorder	6 (15)
		Schizoaffective disorder	1 (2)
		Substance-related disorders	18 (44)
**Axis II diagnosis**			
	No diagnosis		7 (17)
	**Diagnosis**		16 (39)
		Borderline personality disorder	12 (29)
		Personality disorder NOS	4 (10)

^a^NOS: not otherwise specified.

**Table 2:** Baseline demographic and clinical characteristics (N=41).

Characteristics		Values
Sex (female), n (%)		28 (68)
Age (years), mean (SD)		23.4 (3.5)
Hours of Beiwe sensor data (screen-on and accelerometer), mean (SD)		1724 (753)
Beck Depression Inventory scores, mean (SD)		6.96 (8.45)
**Beiwe ecological momentary assessment, mean (SD)**		
	“About how many hours did you actually sleep?”	7.43 (1.03)
	“About what time did you go to bed last night, regardless of the time you actually fell asleep?”	11:28 PM (1.92 hours)
	“What time did you wake up?”	7:41 AM (2.17 hours)
	“How happy versus sad do you feel right now? (1-Very cheerful or happy, 2, 3, 4, 5, 6, 7-Very sad or depressed or unhappy)”	3.12 (1.5)

### Ethical Considerations

All participants provided informed consent for all study procedures. For minors, the parents or guardians, in addition to the minors, provided informed consent. This study was approved by the Institutional Review Board of the University of Pennsylvania (828424).

### Data Acquisition

From the 41 participants, 2972 person-days of sensor data, including accelerometer measures (meters per second squared) for the axes, were obtained from participant smartphones through the Beiwe app, a research platform developed by the Onnela Lab at the Harvard TH Chan School of Public Health [64]. Screen-on events for Android devices were recorded, whereas screen-unlock events for iOS devices were acquired through Beiwe; however, we have referred to both as *screen-on events* in this paper for simplicity. Every morning, participants were also asked about their mood and sleep patterns and quality from the night before via self-report prompts delivered by Beiwe. These questions included sleep duration in hours (“About how many hours did you actually sleep?”), time to sleep (“About what time did you go to bed last night, regardless of the time you actually fell asleep?”), and time to wake (“What time did you wake up?”) and were obtained using self-report questionnaires. The possible time-to-sleep and time-to-wake responses were limited to hour-long intervals. Participants were asked to rank their mood with the following question: “How happy versus sad do you feel right now? (1-Very cheerful or happy, 2, 3, 4, 5, 6, 7-Very sad or depressed or unhappy).” A summary of the demographic and EMA covariates is provided in Table 2. In addition to these questions administered through smartphones, additional measurements were collected at baseline, including the PSQI and the Beck Depression Inventory (BDI) scores [65,66].

### Data Processing

Our analysis goals are 2-fold: (1) to use smartphone sensors and activity data to quantify the strength of each participant’s activity rhythm or routine and subsequently (2) to test for significant associations between demographic variables or self-reported mood outcomes and the strength of activity rhythm or routine. Given our first goal of modeling a participant’s activity rhythm, we leveraged active smartphone use to provide an indicator of activity over the course of the day. From accelerometer data, hourly features were calculated to reflect the magnitude of movement of the smartphone. For each hour of the day, labeled by the time at the end of hour *t*, *X*(*t*) is the mean of the magnitude of phone acceleration over the course of that hour. In addition, *Y*(*t*) is the screen-on count over the course of the hour. However, the periods of dormancy, where *X*(*t*) was unobserved, because of user- and device-related factors such as the phone being powered off, having no cell signal, or being in airplane mode required accelerometer features to be imputed.

**Figure 1:**
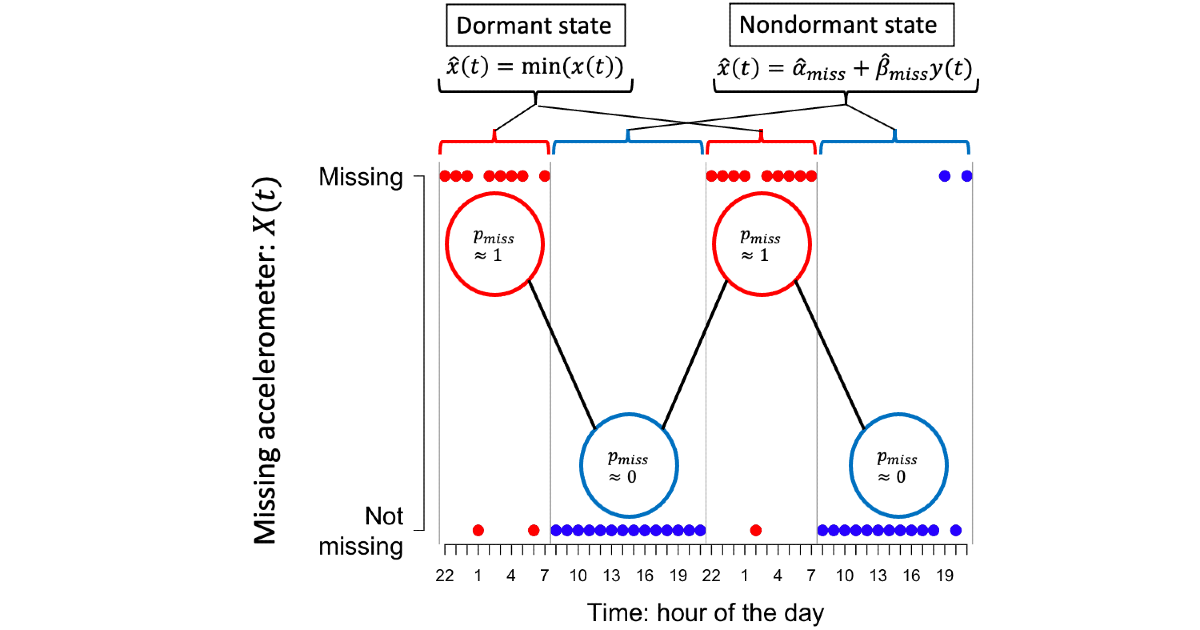
Hidden semi-Markov model to identify periods of dormancy and missing data imputation. First, a hidden semi-Markov was used to identify periods of dormancy, which were characterized by a high proportion of missing accelerometer data, often associated with night-time hours. Hidden semi-Markov allows for a geometric dwell time distribution with Bernoulli outcomes to model the proportion of missingness over consecutive hours. Second, missing mean accelerometer magnitudes X(t) for dormant states were imputed using the minimum (excluding outliers) mean accelerometer magnitudes X(t), whereas missing data for nondormant states were imputed using linear regression.

By considering the characteristics of screen-on events and accelerometer data, we designed a data imputation procedure guided by domain knowledge. Periods of dormancy usually align with periods of low phone use such as night-time hours, have a greater probability of missing accelerometer data *X*(*t*), and were identified using a 2-state hidden semi-Markov model with Bernoulli state-dependent distributions [67]. For example, if accelerometer data are seldom missing over a given window of time and there are many screen-on events over the same period, it is likely that there was significant accelerometer activity despite being missing. Here, screen-on events can be used to impute accelerometer data. In contrast, if accelerometer data are missing and there are no screen-on events, then it is likely that the phone was in a state of dormancy with low accelerometer magnitudes. The periods of dormancy correspond to the 2 latent states in our hidden semi-Markov model imputation. Missing mean accelerometer magnitudes from dormant periods were imputed using the minimum (excluding outliers) mean accelerometer magnitudes *X*(*t*), whereas missing data assigned to the nondormant state were imputed by regressing *X*(*t*) on *Y*(*t*) over all hours where data were completely observed. Ultimately, this led to an imputed *X*(*t*), which we used in the following analyses, with a diagram of the imputation procedure outlined in Figure 1.

The final source of noise in the data was user error, which often occurred when answering questionnaires, such as accidentally selecting 8 PM for wake-up time instead of 8 AM. To avoid these user errors, bedtimes were automatically corrected to be between 5 PM and 4 AM, and wake-up times were corrected to be between 4 AM and 3 PM when such extreme discrepancies occurred that resulted in unrealistic sleep durations that were almost certainly the result of measurement error.

### Activity Rhythm Modeling

Stochastic models are often used to study longitudinal data sets such as the data generated by smartphones, and we opted to use a continuous-time Markov chain framework with the addition of random intercepts representing each hour of the day to model activity rhythms for each participant separately. This choice was driven in part by its ability to account for missing data for which many harmonic analyses were not designed [68]. If an hour of the day has a large random intercept, it represents a higher probability of active phone use during that hour relative to other hours of the day for a specific participant. With this interpretation in mind, a participant with a strong activity rhythm will have hour-of-the-day random intercepts with a large magnitude or, equivalently, with high variability. In addition, the variances of the random intercepts are the test statistics of a mixed-effect ANOVA, where the null hypothesis is that the hour of the day has no effect on the state transition. Phone use, binned into hour-long intervals of activity or rest, was fitted with a 2-state continuous-time Markov chain. By modeling transition rates with an exponential proportional hazard (PH) regression with time-varying covariates, we treated the state labels as latent variables, which correspond to an HMM.

In our HMM, screen-on counts, *Y*(*t*), were characterized by a mixture of 2 state-dependent Poisson distributions with a rest state, *C*(*t*)=2, where *E*[*Y*(*t*)|C(*t*)=2]≈0, and an active state, *C*(*t*)=1, where *E*[*Y*(*t*)|C(*t*)=1]>0. We also incorporated accelerator magnitude averaged over the hour, *X*(*t*), and hour-of-the-day random intercepts (or frailties) in an exponential PH regression used to estimate rates of transitioning from the rest-to-active and active-to-rest states. Random intercepts can be viewed as a penalized effect that the hour of the day has on transition rates and can be interpreted as the activity rhythm. Using our rates and event times, we use Kolmogorov equations to estimate transition probability matrices and construct a 2-state mixed CT-HMM [69]. The transition rates are as follows:

Rest to active: λ_1_(*t*)=exp×(α_1_+β_1_×*x*[*t*]+*b*_1_[*t*]), with *b*_1_(*t*)∼*N*(0,σ_1_^2^) **(1)**

**Figure 2:**
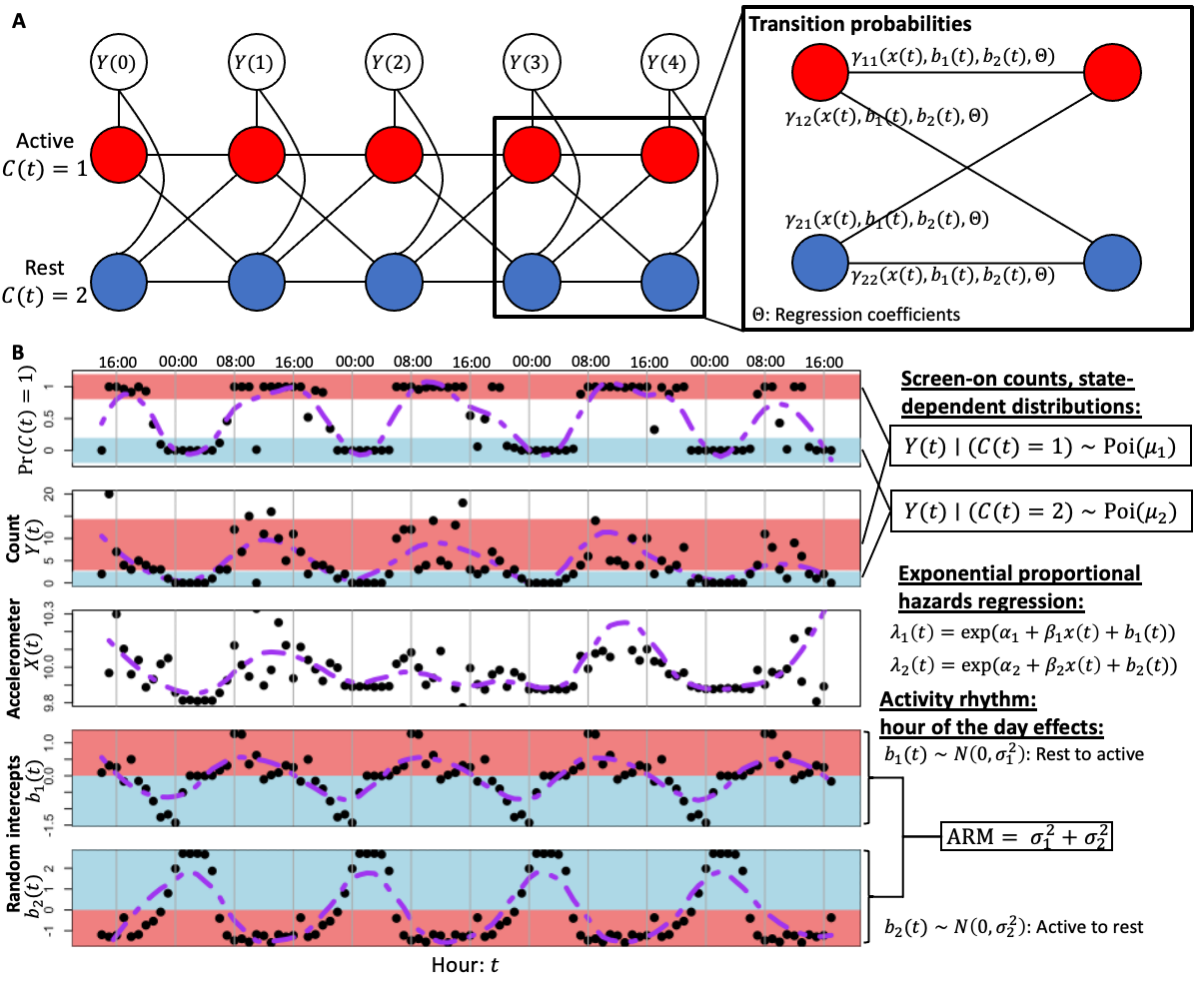
Hidden Markov model framework and the ARM. (A) Graph of the 2-state continuous-time hidden Markov model used to model phone engagement via screen-on counts. Active states are characterized by expected screen-on counts >0, and the rest states are characterized by expected screen-on counts close to 0. Transition rates between rest and active states are modeled with exponential proportional hazard regressions by using 24 hours of the day as random intercepts. (B) Hourly state membership probabilities, state-dependent distribution counts, accelerometer norms, and random intercepts for the hour of the day. We controlled for accelerometer activity in the regression models while estimating activity rhythm with random intercepts. Accelerometer activity is positively correlated with rest-to-active transitions and negatively correlated with active-to-rest transitions. Large hour-of-the-day effects correspond to a regular activity rhythm and resulted in a large ARM. ARM: Activity Rhythm Metric.

Active to rest: λ_2_(*t*)=exp×(α_2_+β_2_×*x*[*t*]+*b*_2_[*t*]), with *b*_2_(*t*)∼*N*(0,σ_2_^2^) **(2)**

where *b*_1_(*t*) and *b*_2_(*t*) are random intercepts for the hour of the day. Subsequently, the corresponding transition rate matrices, *Q*(*t*), are functions of λ_1_(*t*) and λ_2_(*t*). The transition probability matrices are given by the matrix exponential Γ(*t*)=e*^Q^*^(^*^t^*^)^ as the event times are 1-hour increments (Figure 2). In cases where periods of consecutive missing accelerometer data continue over 24 consecutive hours, this constitutes a sufficiently long period of missing data that requires splitting the HMM into 2 segments on either side of the missing interval, where the likelihood of the multiple HMMs can be treated as independent and multiplied together during parameter estimation. The CT-HMM is fitted with the expectation-maximization (EM) algorithm by iteratively solving Θ=(α_1_,β_1_,α_2_,β_2_), *b*_1_(*t*), *b*_2_(*t*), σ_1_^2^, σ_2_^2^ and *Pr*×(*C*[*t*]=*i*,*C*[*t*+1]=*j*) [70-72]. A high frequency of missing data can result in an identifiability problem when fitting the HMM; for example, when an individual seldom uses their phone and the 2 states of the HMM become indistinguishable as the data may not reflect the daily differences in activity rhythms. Seldom phone use results in low screen-on counts and missing accelerometer data, leading to unimodal data streams, even after imputation, where daily differences are difficult to identify. Following this procedure, 7% (3/41) of individuals were omitted from the analysis as their EM algorithms failed to converge because of the much higher than normal frequency of missing data, resulting in a final sample size of 38.

### Comparing Activity Rhythms, Self-reported Sleep, and Depression-Related Variables

After modeling each participant’s activity rhythm via the CT-HMM—random intercepts corresponding to the effect of the hour of the day on the likelihood of rest versus activity—we naturally expect regularity in activity profiles to correspond with large values of the variance of the random intercepts as quantified by σ_1_^2^ and σ_2_^2^. In other words, if a person tends to be active or at rest during the same hours of the day routinely, then the restful hours will have very low random intercepts, with active hours having high random intercepts. Under the mixed-effects ANOVA, the large random intercept variances correspond to rejecting the null hypothesis that the hour of the day has no effect on state transition. This large discrepancy in random intercepts between different hours of the day manifests as large values of σ_1_^2^ and σ_2_^2^. Thus, for each individual, we fit a CT-HMM and sum over these 2 variance terms to obtain what we define as Activity Rhythm Metric (ARM): ARM=σ_1_^2^+σ_2_^2^ (Figure 2).

With quantification of the strength of each participant’s activity rhythm or routine through the ARM, we validate the ARM as a measure of daily rhythm by treating it as the outcome in a linear regression to test for associations with the following self-reported sleep-related covariates: mean nightly sleep duration (mean response of “About how many hours did you actually sleep?”), the baseline sleep duration component of the PSQI, time-to-sleep SD (SD of “About what time did you go to bed last night, regardless of the time you actually fell asleep?”), and time-to-wake SD (SD of “What time did you wake up?”). We would expect a higher ARM to correspond with a stronger routine and therefore with small time-to-sleep or time-to-wake SDs and longer mean sleep duration. For each sleep-related covariate alone, we fit linear regression models controlling for age and sex and compared it with a null model with only age and sex, using a likelihood ratio test (LRT) to test for the association in a 2-sided alternative hypothesis. We used the mean mood response from the Beiwe questionnaire (mean response of “How happy versus sad do you feel right now?”) and BDI as a depression-related measure, with higher mean values corresponding to severe depression. For each depression-related covariate, we tested for an association with the ARM by using the same LRT framework. In addition, we tested the association between age or sex and the ARM while controlling for the others by using the LRT.

## Results

### Relationship With Sleep Duration

**Figure 3:**
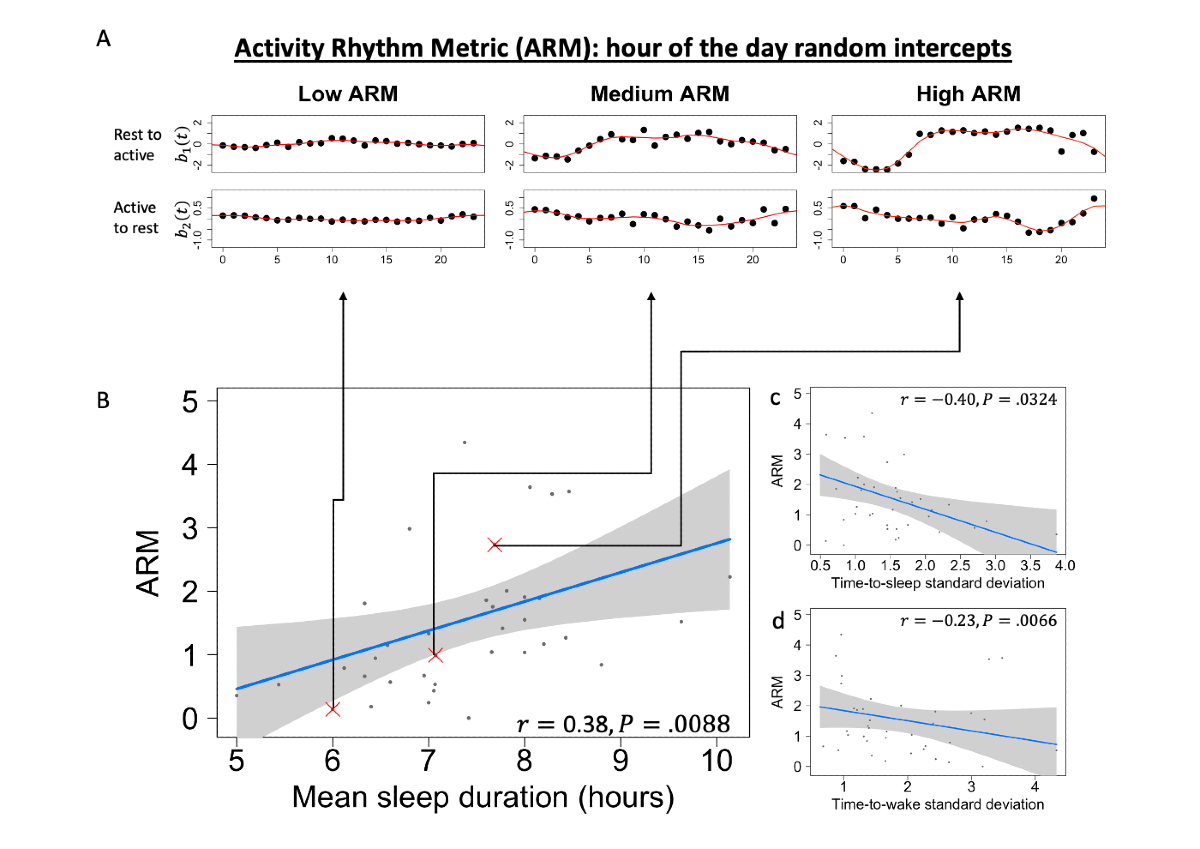
The Activity Rhythm Metric and self-reported sleep. (A) Visualization of 3 example participants with hour-of-the-day random intercepts mapping to low, medium, and high Activity Rhythm Metrics. Random intercepts are the hour-of-the-day effect on state transition: low or high Activity Rhythm Metric score participants have low or high variability in random intercepts. (B) Linear regression models were fit between the Activity Rhythm Metric and sleep-related outcomes across all participants using a likelihood ratio test for and association with each sleep measure while controlling for age and sex. Higher Activity Rhythm Metrics are associated with a longer mean duration of sleep (r=0.38). (C and D) Higher Activity Rhythm Metrics are associated with less variability in self-reported time-to-sleep (r=−0.40) and time-to-wake (r=−0.23) responses.

We found that all sleep-related measures, namely, mean nightly sleep duration, the baseline sleep duration component of the PSQI, time-to-sleep SD, and time-to-wake SD, were marginally significantly (*P*<.05) associated with the ARM (Figure 3). Association tests included only individuals with corresponding self-reported outcomes. Notably, continuous daily administration of sleep surveys may increase the patient burden in a study, highlighting the advantages of passive data collection methods. We found that individuals with a higher ARM are more likely to have longer sleep duration, with an hourly increase in mean sleep duration corresponding to a 0.4 increase in the ARM. The mean nightly sleep duration from the Beiwe questionnaires (“About how many hours did you actually sleep?”) captures the same information as the baseline sleep duration component of the PSQI, which was also significantly (*P*<.001) associated with the ARM and had the same direction of effect. Considering sleep duration as a component of sleep quality, these findings suggest that the ARM was positively correlated with sleep quality. In other words, a stronger and more consistent routine, as measured passively through smartphone use, corresponds to better sleep quality.

### Relationship With Self-reported Sleep and Wake Times

The questionnaires captured by Beiwe through smartphones prompted participants to report time to sleep and time to wake, which have been used to measure the regularity of activity rhythms in other contexts [73]. We expected that the high variance in self-reported time-to-sleep and time-to-wake responses would correspond to irregular routines. This finding is consistent with the relationship between the ARM and variability in self-reported sleep or wake timing. In particular, we found that the ARM is significantly correlated with the SDs of time-to-sleep (“About what time did you go to bed last night, regardless of the time you actually fell asleep?”) and time-to-wake (“What time did you wake up?”) responses (Figure 3). A unit increase in time-to-sleep SD corresponds to a 0.52 decrease in the ARM, and a unit increase in time-to-wake SD corresponds to a 0.48 decrease in the ARM. Thus, our proposed ARM measure passively captures many of the same routine-related signals as traditional survey-based metrics while avoiding the high burden of daily self-reporting, which is otherwise necessary to collect data on the variability of the sleep schedule.

### Relationship With BDI and Self-reported Mood

**Figure 4:**
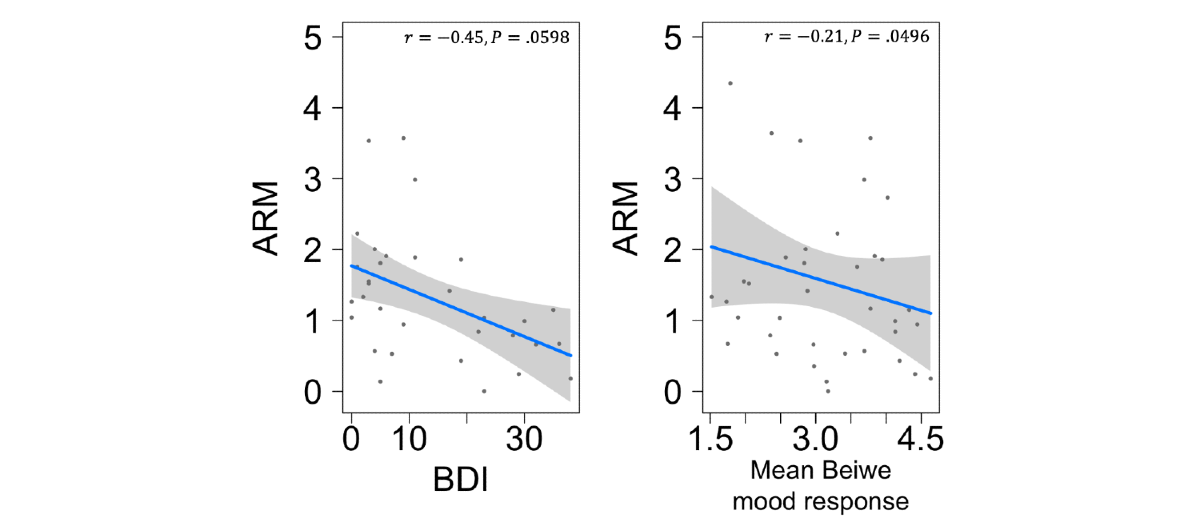
Activity rhythm and its relationship with mood and depression. In total, 2 mood or depression-related self-reported outcomes were compared with the ARM: (1) The BDI was recorded for 32 participants, and (2) the mean response from the Beiwe smartphone mood question: “How happy versus sad do you feel right now? (1-Very cheerful or happy, 2, 3, 4, 5, 6, 7-Very sad or depressed or unhappy).” Linear regression models were fit by using a likelihood ratio test while controlling for age and sex. ARM and BDI have a negative correlation (r=−0.45, *P*=.06). ARM and mean response from the Beiwe mood survey have a negative correlation (r=−0.21, *P*=.049). ARM: Activity Rhythm Metric; BDI: Beck Depression Inventory.

In a variety of clinical populations, there is evidence of a relationship between depression-related metrics and irregular routines, where the quantification of regular rhythms can be used to assess treatment efficacy [27]. In line with this, we found that the ARM was negatively correlated with the BDI (Figure 4); that is, irregular activity rhythms were associated with higher BDI. Of note, only the response to the Beiwe mood question (“How happy versus sad do you feel right now?”) was marginally associated with the ARM (*P*=.049); however, the direction of the associations was intuitive, albeit borderline significant (Figure 4). Of note, 15% (6/41) of additional participants had missing BDI scores. The average ARM for the participants with missing BDI was 2.86, whereas it was much lower (1.31) for participants who had recorded the BDI; it is likely that the missing BDI information, if observed, would help increase the precision of the association and increase its strength. The difference between the average BDI values of these 2 groups suggests that informative missingness may have diminished statistical power.

### Relationship With Age and Sex

**Figure 5:**
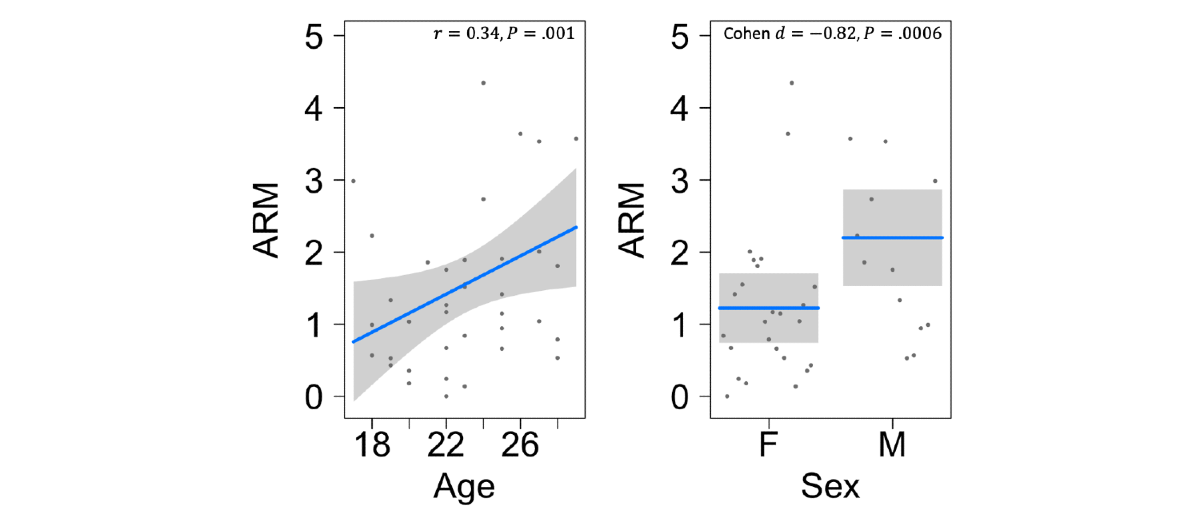
Activity rhythm and its relationship with age or sex. Linear regression models were fit by using a likelihood ratio test for age or sex each while controlling for the other covariate. Age is positively correlated with ARM (r=0.34, *P*=.001), and male participants have higher ARMs (Cohen d=−0.82, *P*<.001). ARM: Activity Rhythm Metric.

In addition to the ARM being associated with sleep-related measures of duration and variability in time-to-sleep and time-to-wake responses, we found that the ARM was associated with age in a manner similar to previous regularity of rhythm studies, although previous studies examined different study populations [73,74]. We found a positive correlation between age and the ARM (*P*=.001); a year increase in age corresponds with a 0.18 increase in the ARM, meaning that older individuals tend to have more regular activity rhythms and routines (Figure 5). In addition, our analysis of the ARM suggests that there is a significant (*P*<.001) sex-based difference in the regularity of a participant’s activity rhythm (Figure 5). We expect a 1.31 increase in the ARM of male participants in our sample relative to that of female participants.

## Discussion

### Principal Findings

The regularity of daily routine, as measured through the ARM—a quantification of routine based solely on passively collected smartphone data— was found to be significantly associated with a variety of demographic, mood, and sleep-related measures. We developed a CT-HMM that allows for an hour-of-the-day effect on state membership (active vs rest). Using the variance of the hour-of-the-day random intercepts to represent the strength of the routine, we constructed an ARM and found it to be associated with the SD in self-reported time-to-bed and time-to-wake responses on a night-to-night basis. These findings validate the ARM as a quantification of the strength of routine, which can be used as an outcome in studies aimed at improving mental health by increasing regularity in routine. Furthermore, the ability to calculate the ARM using only passively collected smartphone data provides a crucial advantage relative to the traditional reliance on self-report to dynamically quantify routine. This can provide a low-burden alternative that can easily be deployed at scale, even in studies with long follow-up durations, as passively collected data are not susceptible to the same survey fatigue, which makes long-term follow-up a challenge in studies that rely heavily on self-report.

### Comparison With Prior Work

The direction of associations for the ARM is aligned with existing metrics such as the SRM. The SRM is a diary-based metric that has helped inspire our ARM definition and approach, where both are calculated from intraindividual routine variations, and higher scores correspond to regular routines [20,21]. The ARM evaluates active or rest states timing akin to calculating sleep or wake variability in time from data obtained through a survey or diary. Both the ARM and SRM are highly influenced by variances in the timing of habitual daily behaviors, such as time to sleep and time to wake, where a high variance corresponds to a low score. Our findings showed that a low ARM is associated with high variability in time-to-sleep and time-to-wake self-reported responses, indicating that the ARMs are correlated with information that would otherwise be obtained through diary-based metrics.

Our results, which link the ARM to sleep duration, reinforce some findings derived from diary-based methods. Monk et al [73,75] found that the SRM was negatively correlated with better sleep quality, as measured by the PSQI. Similar to the SRM, we found that the ARM tends to increase with age, with older participants tending to have greater regularity in their routines [73,74]. Our analysis also showed greater regularity in activity rhythms in male participants, which aligns with the findings by Monk et al [67], despite some studies showing conflicting data on sex-based differences in the SRM [20,73,76,77]. Although the ARM is inspired by the SRM, we noted that the ARM fundamentally represented a narrower scope than the SRM by only focusing on a person’s activity and with no direct measurement of social behavior.

We expected that the ARM would tend to decrease with higher depressive symptoms, reflecting the established relationship between stronger routines and milder depression [27]. Although the same direction of effect was shown in our sample (Figure 4), we lacked the sample size to achieve more than marginal statistical significance in this association. Similar psychiatric studies had access to larger cohorts of participants and healthy controls, which we lacked in this study, and would greatly improve the statistical power [20,26]. An important next step is to repeat these analyses with a larger sample size to validate which relationships hold. Consequently, the potential to quantify routines using only passively collected data may be an informative and actionable digital biomarker with respect to clinically relevant outcomes such as depression.

### Limitations

Our analysis has limitations typical of a retrospective study—a secondary analysis with limited data. The absence of a control group could have affected the statistical power. For example, in a case-control study, we expected the control group to have high ARMs and low BDI scores. Data from a control group would be high leverage points in regression analysis in certain situations and would increase the effect size. In addition, this study was limited to a small age range. An in-depth analysis of regularity was not anticipated during the initial recruitment of the study cohort; subsequently, diary-based regularity metrics were not available for a direct comparison with ARMs. In summary, our analysis explored connections between routine activity rhythms and several clinical covariates; however, the validation of relationships and generalization to broader populations are left to future studies with prospective designs. In addition, differences between device hardware and operating systems could introduce heterogeneity in the data and should be accounted for in mHealth studies. For example, iOS acceleration is normalized by the g-force constant, whereas Android acceleration is not. Our individual-specific PH regressions, as discrete class models, were not affected by a scale difference in the covariates, and the g-force constant was absorbed by the coefficient estimate.

Although the use of passively collected smartphone data successfully reduces participant burden, there are some hurdles in this type of data collection. In certain cases, many clinical populations [78], including ours, are willing to share sensor data despite privacy concerns. In addition, sharing ARM-like summary statistics rather than the entire collection of sensor data may attenuate the privacy concerns of clinicians, allowing studies to increase the sample size. However, accounting for the missing data of various missing mechanisms remains paramount, and there is a lack of methods available to handle missingness when it is associated with the outcome of interest (missing not-at-random). Although we proposed a simple domain-based approach that takes into account missingness because of a lack of phone engagement induced by diurnal patterns, additional sensitivity analyses using different imputation procedures are necessary to completely understand the effect of missing data in mHealth studies.

In addition, identifiability is an important concern in mixed-effect modeling. A sufficient sample size for each hour must be available for modeling random intercepts. HMMs, which are models with many parameters, require large sample sizes or strong signals for parameter estimation. Although our EM algorithm failing to converge indicates an identifiability concern with the underlying data, other criteria may be used for the explorative analysis of data before HMM fitting. In participants with limited data, the hour-of-the-day effects must be pronounced to fit the random intercept model. In other words, sufficient evidence is needed to detect hour-of-the-day effects, either adequate sample size or the strength of association between hour-of-the-day and state membership. In our case, a lack of engagement with the study phone leading to unimodal data streams is an important consideration related to model fit but is addressed by our failure of convergence criterion. Proper use of study phones is an important prerequisite for mHealth studies, and exploring steps for filtering out individuals based on data quality is an important component of the mHealth study design. Data quality, both handling of missing data and proper use of the study device, remains a paramount concern in mHealth studies. As a result, we look to evaluate the many relationships uncovered by our analyses in future work involving different study cohorts and different methods for handling missing data.

### Potential Future Directions

Our novel CT-HMM framework could easily be adapted to incorporate additional information to improve its ability to quantify diurnal activity patterns and routines. For example, we can extend the univariate outcome, *Y*(*t*), to a multivariate joint probability distribution that also considers longitudinal GPS data. Although we modeled a parsimonious representation of daily routines, a common prompt in SRM diaries is the time of starting work, which can be ascertained from GPS location. By incorporating GPS data and increasing the number of states in our CT-HMM beyond the 2 rest or active states used in this study, we can model a wider range of routine behaviors and participant states.

Alternative HMM formulations that allow latent states to represent more than just rest or active states, such as symptom severity, would make the expected timing of state transitions clinically relevant. Many mixed-effect HMMs use a logit link or logistic regression to model transitions between states, where coefficients can be interpreted as odds ratios [60,61]. We elected to use a PH model, where the coefficients can be interpreted as hazard ratios. Although the interpretation of the signs of the coefficients is similar across both models, under the PH model, the expected event time (or time until state transition) can be calculated as 1/λ(*t*). This expected event time is intuitively important as it allows for the prediction of state changes in an individual, which can be used to prompt a mHealth intervention in the context of an HMM framework where latent states could represent, for example, manic, or depressed states in an individual with bipolar disorder.

### Conclusions

The previous generation of diary-based metrics comparable with the ARM are limited by self-report, which requires a high burden on the participant, underscoring the potential of mHealth solutions. However, the identifiability of complex models for mHealth data should be taken into consideration during study design. We estimated each participant’s activity rhythm and corresponding strength of routine by calculating participant-specific hour-of-the-day random intercepts in a novel CT-HMM modeling framework that dictated consistency in phone activity over the course of the day for each participant. By using passively collected smartphone use and accelerometer data, the CT-HMM was able to identify rest-activity states and the effect of the hour of the day on the likelihood of being active or at rest, which we used to construct the ARM, and found it to be associated with a variety of demographic, sleep, and mood or depression variables. We validated the ARM relative to self-reported nightly sleep-wake cycles and found that the ARM was correlated to variability in sleep or wake times from Beiwe surveys. It is important to note that additional follow-up studies are necessary to validate our ARM covariate relationships. Our primary analyses suggest that the ARM is a promising alternative to previous diary-based metrics, which are often used to assess treatment efficacy.

## Abbreviations

ARM: Activity Rhythm Metric
BDI: Beck Depression Inventory
CT-HMM: continuous-time hidden Markov model
EM: expectation-maximization
EMA: ecological momentary assessment
HMM: hidden Markov model
LRT: likelihood ratio test
mHealth: mobile health
PH: proportional hazard
PSQI: Pittsburgh Sleep Quality Index
SRM: Social Rhythm Metric
